# Bacterial colonization of handheld devices in a tertiary care setting: a hygiene intervention study

**DOI:** 10.1186/s13756-019-0546-y

**Published:** 2019-06-06

**Authors:** Pascal M. Frey, Grischa R. Marti, Sara Droz, Mirjam de Roche von Arx, Franziska Suter-Riniker, Drahomir Aujesky, Silvio D. Brugger

**Affiliations:** 1 Department of General Internal Medicine, University Hospital Bern (Inselspital), University of Bern, 3010 Bern, Switzerland; 2 Department of Infectious Diseases and Hospital Epidemiology, University Hospital Zurich, University of Zurich, Zurich, Switzerland; 3 Institute for Infectious Diseases, University of Bern, Bern, Switzerland; 4 Department of Internal Medicine, Hospital of Thun, Thun, Switzerland

**Keywords:** Tablet computers, Bacterial colonization, Hygiene intervention

## Abstract

**Background:**

Tablet computers are increasingly being used in hospital patient care and are often colonized with important human pathogens, while the impact of disinfection interventions remains controversial.

**Method:**

In a prospective hygiene intervention study we consecutively sampled tablet computers exclusively used in a high-resource general internal medicine tertiary care setting with high routine hygiene measures. Our aim was to examine the change in colonizing bacteria on tablet computers before and after the introduction of a mandatory twice daily tablet disinfection intervention. Microbial identification was performed by conventional culture, and the association of bacterial colonization with the intervention was investigated using logistic regression.

**Results:**

In a total of 168 samples we identified colonizing bacteria in 149 (89%) of samples. While the most commonly identified species were normal skin bacteria, *Staphylococcus aureus* found in 18 (11%) of samples was the most frequent potential pathogen. We did not detect any Enterococci or Enterobacteriaceae. The disinfection intervention was associated with substantially less overall bacterial colonization (odds ratio 0.16; 95%-CI 0.04–0.56), while specific colonization with *Staphylococcus aureus* was only slightly decreased (odds ratio 0.46; 95%-CI 0.16–1.29).

**Conclusion:**

Our results indicate that a twice daily disinfection can still substantially reduce bacterial colonization of in-hospital tablet computers used in a high-resource and high hygiene setting.

## Introduction

Handheld devices like tablet computers, smartphones and other digital equipment have become a daily feature of medical work. Several studies have investigated bacterial colonization on cell phones in cross-sectional designs and found a high prevalence of coagulase-negative Staphylococci, while among bacteria considered as pathogens *Staphylococcus aureus* was the most frequent [1]. Prevalence of MRSA (methicillin-resistant *S. aureus*) has been reported to be up to 10% depending on sample site [2]. Further pathogens such as *Enterococcus* spp., *Pseudomonas aeruginosa*, *Escherichia coli* and *Klebsiella* spp. have been reported to colonize cell phone surfaces [2–4]. An analysis of health care workers‘ cell phones revealed a prevalence of 96.2% for bacterial colonization with 14.3% of mobile phones being colonized with bacteria that are known to cause nosocomial infection [3]. However, only 14 of 25 studies investigating the use of decontamination methods reported a significant decrease in bacterial colonization on a multitude of devices used in different inpatient and outpatient settings. Therefore, the effect of disinfection interventions on bacterial colonization of tablet computers used by healthcare professionals exclusively in a hospital setting still remains controversial [1].

In the year 2013, the Department of General Internal Medicine, Bern University Hospital, Inselspital, introduced iPads® for bedside use during late and night shifts for mobile access to patient data, laboratory values, X-ray images and findings, medical literature and guidelines. This provided an excellent opportunity to investigate the microbial composition found on tablet computers used in a tertiary hospital setting and determine microbial colonization changes during a disinfection intervention. We hypothesized that an additional disinfection measure would further decrease bacterial colonization of tablet computers.

## Methods

### Study design, setting and sampling

For this hygiene intervention study, we swabbed hospital tablet computers (iPad® 2^nd^ Generation) routinely carried by physicians on shift duty after late and night shifts from June 2013 to February 2014. Physicians were using the tablets during inpatient bedside care at the Department of General Internal Medicine of the Bern University Hospital, a tertiary care teaching hospital.

The study was composed of two phases: a pre-intervention phase with routine hygiene measures, and an intervention phase where additional device disinfection was initiated. During the pre-intervention phase users of the tablet computers were not informed about the study to ensure unbiased handling of devices according to routine hand hygiene procedures already in place. For the intervention phase from December 2013 to February 2014, a disinfectant station with 70% ethanol and an instructional poster was put up at the tablet computer charging station, instructing doctors to wipe down their used iPad® with the disinfectant before putting it back into the charging dock at the end of their shift. Although the disinfection process was communicated as mandatory, there was no monitoring or enforcement of doctors’ compliance.

Consecutive sampling was performed for each the pre-intervention and intervention phase by the investigators in the morning after conclusion of shift work. Swabbing was done using sterile eSwabs (Copan, Brescia, Italy) wetted with transport medium contained in eSwabs covering front and back of the tablet computer. To ensure standardized swabbing, precise instructions were created and used by all authors. Gloves were worn during swabbing, and surfaces of the devices were cleaned with dry wipes after sample collection to clean off residual swabbing liquid to keep users from becoming aware of the devices being sampled.

### Microbial culture

Swabs were streaked out onto CSBA, CNA and MacConkey agar plates: CSBA was used for cultivation of non-fastidious and fastidious microbes as a screen for overall growth; CNA was used for selection of Gram-positivebacteria (i.e., Staphylococci, Streptococci, Enterococci, etc.); and MacConkey Agar was used for the selection of Gram-negative bacteria (i.e., Enterobacteriaceae). Agar plates were incubated at 37 °C in 5% CO_2_ air for a maximum of 5 days.

Species identification was performed according to morphological properties: type of hemolysis on CSBA/CNA (e.g., true hemolysis for *S. aureus* or alpha-hemolysis for certain Streptococci) and color change on MacConkey Agar (e.g., lactose fermenters such as *E. coli* turning red). Further, we used biochemical reactions for catalase, coagulase and hemolysis tests for the primary identification of *S. aureus*; however, final species identification was confirmed using a Bruker microflex LT MALDI-TOF-MS (MALDI Biotyper) [5].

Subcultures were done - if indicated - for further microbial identification according to biochemical or morphological properties and MALDI-TOF-MS.

In case of *S. aureus* identification, we tested for susceptibility to Penicillin G, Oxacillin, Gentamicin, Cotrimoxazol, Tetracyclin, Clindamycin, Erythromycin and Vancomycin. Oxacillin-resistant strains were considered as MRSA.

During the process of microbial identification potential human pathogens like *S. aureus* were reported separately, while other members of the skin bacterial microbiota such as non-*S. aureus* Staphylococci, Corynebacterium spp. and *Cutibacterium* species were not further distinguished but summarized as “normal skin bacteria”. Accordingly, bacteria of the upper respiratory microbiota were summarized as “normal respiratory bacteria”.

### Statistical analyses

The primary outcome measure was defined as the total reduction of samples with qualitative bacterial growth in agar-based culture assays before and during the disinfection intervention. The number of samples with bacterial growth before and after introduction of the disinfection intervention were compared using chi-squared tests. Associations between the intervention phase and bacterial colonization were analyzed using univariate logistic regression.

All analyses were performed using STATA 14 for Windows (STATA Corp, College Station, Texas).

## Results

### Microbial culture growth

From overall 168 samples taken throughout the study period (84 routine measures and 84 intervention phase), we observed bacterial growth in a total of 149 samples. The most frequently culture-detected bacteria were normal skin bacteria (in 128 [76%] of samples), followed by normal respiratory bacteria (in 24 [14%] of samples).

**Fig. 1:**
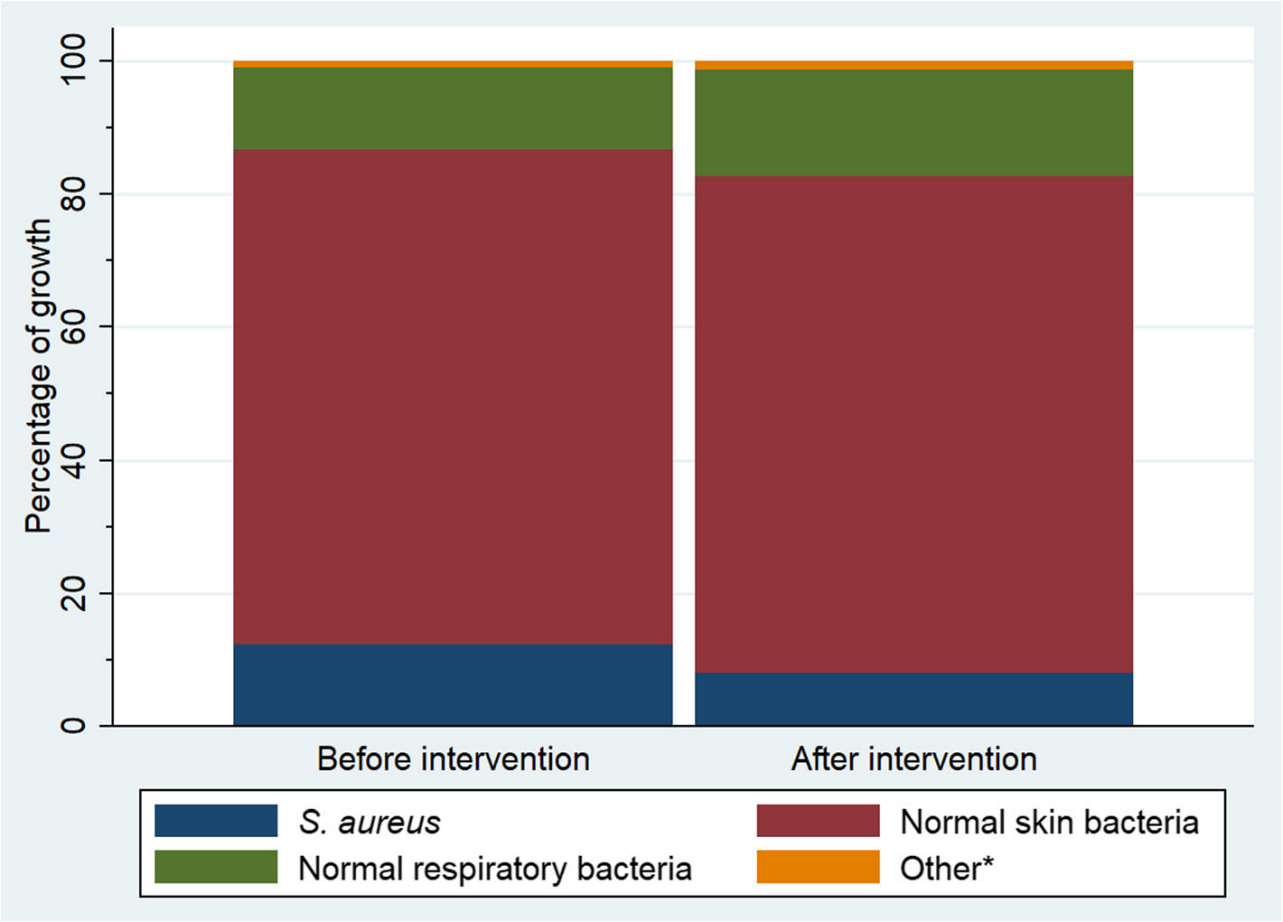
Proportional bacterial growth before and after the disinfectant intervention. Although after the intervention the number of samples with *S. aureus* decreased from 14 to 7%, there was no evidence this change is beyond chance (*p* = 0.13). Percentage of growth is given to the total of bacterial types found before and after the intervention, respectively. * *Streptococcus milleri* identified in one sample before intervention and *Bacillus* spp. in one sample after intervention

While 81 (96%) samples showed bacterial growth before the disinfectant intervention, only 68 (81%) samples were colonized after the intervention (Table 1). Colonization with *S. aureus* decreased from 12 (14%) samples before the intervention to 6 (7%) samples after the intervention, and no methicillin resistant *S. aureus* (MRSA) were detected. The overall proportional composition of bacteria from culture remained similar after the disinfectant intervention (Fig. 1). Neither during the routine hygiene phase nor after the disinfectant intervention could we identify *Enterococcus* spp., *Pseudomonas aeruginosa*, *Escherichia coli* or *Klebsiella* spp. in any of the samples.

**Table 1:** Bacteria identified in culture before and after the disinfectant intervention

	Overall	Pre-intervention	During intervention	*p*-value^a^
		n (%)^b^		
*S. aureus*		12 (14)	6 (7)	0.13
Normal skin bacteria^c^		72 (86)	56 (67)	< 0.01
Normal respiratory bacteria^d^		12 (14)	12 (14)	> 0.99
Others^e^		1 (1)	1 (1)	–

^a^ *p*-values were derived from chi-squared test and the result omitted (−) when sample size < 5.

^b^ n is referring to the total number of bacterial species found, while % is giving the number of species to the denominator of total samples (168). Before and after intervention eras both comprised of 84 samples each, some of which contained multiple types of bacteria. The surplus when numbers or percentages are added up reflects the extent of multiple species per sample.

^c^ Skin colonizing bacteria, excluding *S. aureus*

^d^ Common bacteria known to colonize the respiratory tract

^e^ Comprising of *Streptococcus milleri* identified in one sample before intervention and *Bacillus* spp. in one sample after intervention

### Association between the intervention and bacterial colonization

Tablet computers sampled during the intervention study were substantially less likely to contain any bacteria (odds ratio 0.16; 95% confidence interval 0.04 to 0.56, *p* <  0.01). A similar effect could be seen for *S. aureus* (odds ratio [OR] 0.46; 95% confidence interval [CI] 0.16 to 1.29) and normal skin bacteria (OR 0.33; 95% CI 0.16 to 0.71), but not for normal respiratory bacteria (OR 1; 95% CI 0.42 to 2.37).

## Discussion

In this hospital hygiene intervention study, we determined bacterial colonization of tablet computers by microbial culture, and found a twice daily disinfection intervention to be associated with an overall substantial decrease of bacterial colonization of tablet computers routinely used in a tertiary hospital setting. This finding, including its effect, is consistent with a previous study in an inpatient general internal medicine setting [6].

The main bacterial species discovered on tablets in our study were *S. aureus* and other normal skin bacteria, which is also in accordance with what has been reported previously [1].

The small proportion of *S. aureus* decreased by half after introducing the disinfection intervention, although the total sample size for *S. aureus* was low, and this change might have been observed by chance. All of the detected *S. aureus* were methicillin susceptible, which is likely a representation of the low MRSA prevalence of 8% in Switzerland in 2014 [7], with a further decline to 4.4% in 2017 [8].

In contrast to previous studies [2–4], we did not detect any Enterobacteriaceae or Enterococci, most likely due to the high level of routine hand hygiene measures in this high-income and high-resource setting.

Our study has several strengths. First, we swabbed tablet computers that were used exclusively in the hospital and thus could characterize bacterial colonization without direct household contamination, and were able to evaluate an intervention in a closed hospital environment. Second, the non-enforcement of hygiene measures during the intervention phase was a pragmatic approach that allowed us to evaluate the effectiveness of the intervention in a real-life setting.

This study also has several limitations. First, due to the unexpectedly low prevalence of *S. aureus* or other important pathogens previously found on mobile phones with usage outside the hospital setting, the study was underpowered to detect an effect of the intervention on these bacteria. Second, the sequential design of the pre-intervention and intervention phases makes this study susceptible to a bias by unmeasured confounders, such as a change in shift physicians with different unmeasured chronic colonization with pathogens like *S. aureus*. However, even though the shift staff may have changed, there was no major change in routine hygiene or hospital employment between the two study periods.

## Conclusion

The results from this hospital hygiene intervention study indicate that a twice daily disinfection routine could suffice to substantially decrease bacterial colonization on in-hospital handheld devices. However, to give a thoroughly informed recommendation, a further ascertainment of the effect of such an intervention on important nosocomial pathogens using a large-scale cluster randomized controlled trial would be needed. Further research should also focus on correlating device colonization data with clinically isolated pathogens from hospital acquired infections.

## Data Availability

The datasets used and analyzed for our study are available from the corresponding author upon reasonable request.

## Abbreviations

CI: Confidence interval
MRSA: Methicillin resistant *S. aureus*
OR: Odds ratio
*S. aureus*: *Staphylococcus aureus*
